# A Phase 1 Dose-Escalation Study of the Cardiac Myosin Inhibitor Aficamten in Healthy Participants

**DOI:** 10.1016/j.jacbts.2022.04.008

**Published:** 2022-08-10

**Authors:** Fady I. Malik, Laura A. Robertson, Danielle R. Armas, Edward P. Robbie, Anna Osmukhina, Donghong Xu, Hanbin Li, Scott D. Solomon

**Affiliations:** a Research and Development, Cytokinetics, Inc, South San Francisco, California, USA; b Celerion, Inc, Tempe, Arizona, USA; c Certara, Inc, Menlo Park, California, USA; d Department of Medicine, Brigham and Women’s Hospital, Boston, Massachusetts, USA

**Keywords:** aficamten, cardiac myosin inhibitor, hypertrophic cardiomyopathy, LV contractility, phase 1, AE, adverse event, AUC_24_, area under the plasma concentration–time curve from time 0 to 24 hours, C_max_, maximum plasma drug concentration, CV%,, percent coefficient of variation, CYP, cytochrome P450, CYP2D6-PM, cytochrome P450 2D6 poor metabolizer phenotype, DLRC, Dose Level Review Committee, ECG, electrocardiogram, HCM, hypertrophic cardiomyopathy, LV, left ventricle, LVEDV, left ventricular end-diastolic volume, LVEF, left ventricular ejection fraction, LVESV, left ventricular end-systolic volume, MAD, multiple ascending dose, PD, pharmacodynamic, PK, pharmacokinetic, QTcF, QT interval corrected for heart rate using Fridericia’s formula, SAD, single ascending dose, TEAE, treatment-emergent adverse event

## Abstract

This phase 1, randomized, double-blind, placebo-controlled study of aficamten (formerly CK-3773274) in healthy adults identified a pharmacologically active range of doses and exposures. At doses that were pharmacologically active (single doses of ≤50 mg or daily dosing of ≤10 mg for 14 or 17 days), aficamten appeared to be safe and well tolerated. Adverse events were generally mild and no more frequent than with placebo. Pharmacokinetic assessments showed dose proportionality over the range of single doses administered, and pharmacokinetics were not affected by administration with food or in otherwise healthy individuals with a cytochrome P450 2D6 poor metabolizer phenotype. (A Single and Multiple Ascending Dose Study of CK-3773274 in Health Adult Subjects; NCT03767855)

## Highlights

• Certain genetic hypertrophic cardiomyopathies may result from hypercontractility of cardiac muscle, caused by pathogenic variants in genes encoding proteins of the cardiac sarcomere.
• Aficamten (formerly CK-3773274) is a small-molecule selective inhibitor of the cardiac myosin ATPase, which reduces the contractility of cardiomyocytes in vitro and decreases measures of ventricular contractility in animal studies.
• In this first-in-human, phase 1 study in healthy adults, aficamten was well tolerated; adverse events were generally mild and comparable in frequency to those seen with placebo.
• Aficamten demonstrated dose-proportional pharmacokinetics with a half-life of 75 to 85 hours.
• Pharmacodynamically active doses of aficamten decreased left ventricular ejection fraction from baseline in a concentration-dependent manner, informing the design of a phase 2 trial in patients with hypertrophic cardiomyopathy.

## Visual Abstract

**Figure:**
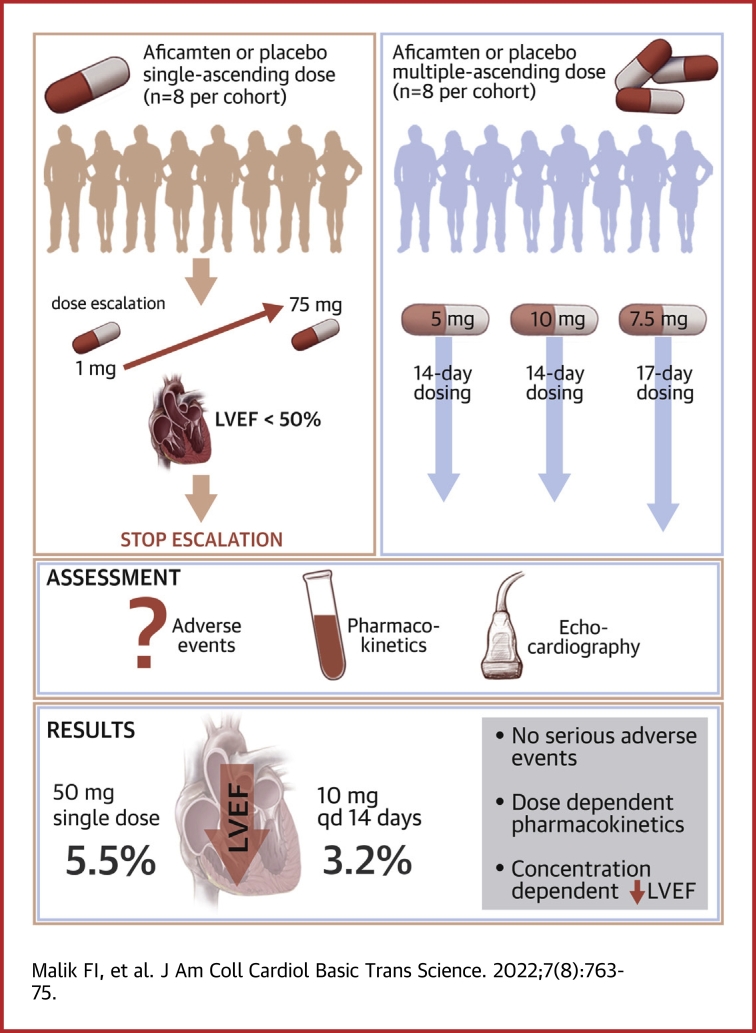

Hypertrophic cardiomyopathy (HCM) is characterized by unexplained cardiac hypertrophy, a nondilated left ventricle (LV), and a normal or increased ejection fraction.^1^ HCM is an important cause of sudden cardiac death, particularly in young adults and adolescents,^2^ and it may be associated with syncope,^1^ heart failure,^3^ and atrial fibrillation, which in turn increases the risk of stroke.^4^ HCM is inherited in an autosomal dominant pattern and is reported to be one of the most common inherited heart conditions, although prevalence estimates for clinically evident disease are lower.^5^^,^^6^

Pathogenic sequence variants in multiple genes can result in HCM, with most of these genes encoding sarcomere-associated proteins^1^^,^^7^ that are responsible for generating and regulating cardiac muscle contraction. These sequence variants produce changes in protein function, leading to a hypercontractile state of the sarcomere, and initiating a series of secondary effects at the cellular and organ levels,^1^ such as development of myocyte disarray, interstitial fibrosis, and cardiac hypertrophy—phenotypic hallmarks of HCM.^1^^,^^7^^,^^8^ Therapeutic correction of the contractile phenotype may limit the adverse clinical outcomes in patients with HCM.^9^ Cardiac myosin inhibitors might therefore have the potential to reduce hypercontractility, impaired relaxation, and adverse LV remodeling in patients with HCM,^10^^,^^11^ thus representing a targeted approach to treating HCM. Therapeutic potential has been demonstrated by mavacamten, a small-molecule selective inhibitor of cardiac myosin that first entered clinical studies in 2015 and has received regulatory approval from FDA^12^ following completion of the phase 3 clinical trial, EXPLORER-HCM (Evaluation of Mavacamten in Adults With Symptomatic Obstructive Hypertrophic Cardiomyopathy).^11^ The trial showed that mavacamten was superior to placebo for improving exercise capacity and health status in patients with obstructive HCM.^11^ Mavacamten also reduced the LV outflow tract pressure gradients that are a hallmark of obstructive HCM, the most common form of HCM. Dosing was guided by echocardiography and plasma concentrations of mavacamten, with dose adjustments occurring after at least 8 weeks of treatment at the prior dose. Prohibited medications included cytochrome P450 (CYP) 2C19 inhibitors (eg, omeprazole or esomeprazole), strong CYP3A4 inhibitors, or St. John’s wort (a CYP3A4 inducer).

Aficamten (formerly CK-3773274) is an investigational small-molecule selective inhibitor of cardiac myosin^13^ discovered following an extensive chemical optimization program that was conducted with careful attention to therapeutic index and pharmacokinetic (PK) properties.^13^

Aficamten reduces the number of active actin-myosin cross bridges during each cardiac cycle and consequently suppresses myocardial hypercontractility. In preclinical models, aficamten reduced myocardial contractility by binding directly to cardiac myosin at a distinct and selective allosteric binding site, thereby preventing myosin from entering a force-producing state.^13^ The inhibition of contractility was achieved without disrupting calcium transients within the myocyte.^13^ Preclinical in vivo studies have shown that aficamten reduces ventricular contractility in a dose- and exposure-dependent manner in the rat and dog^13^ and in the R403Q murine model of HCM (unpublished data, 2019, on file at Cytokinetics).

Although mavacamten and aficamten are both cardiac myosin inhibitors, the preclinical profile of aficamten differs from that of mavacamten in certain aspects. Characterization of mavacamten shows that it requires 6 weeks to reach a steady state, and its pharmacologic effect can be prolonged upon discontinuation of the drug.^14^^,^^15^ For aficamten, the preclinical characterization suggests that the half-life of aficamten in humans could enable once-daily dosing, attainment of steady-state plasma concentrations within 2 weeks, and reversibility following dose reduction or discontinuation of dosing.^13^ A comparison in rats and dogs suggested that the exposure-response relationship of aficamten was shallower than that of mavacamten by roughly 3-fold.^13^ For a drug class that is individually titrated to effect, the PK and steepness of the exposure-response relationship are important properties, with the pharmacokinetics relating to the time to onset/offset of effect and the exposure-response relationship relating to the onset of effect as the dose increases. The properties of aficamten may yield benefits to patients in terms of efficacy, safety, and ease of use, and thus it was advanced into humans.

This first-in-human, phase 1, randomized study examined the safety, tolerability, and PK characteristics of aficamten in healthy adults. Pharmacodynamic (PD) properties were also evaluated, using comprehensive echocardiography to assess and quantify the effect of aficamten on cardiac function. Additional objectives were to examine the impact of the CYP2D6 poor metabolizer (CYP2D6-PM) phenotype and the effect of administration with food on the PK of aficamten.

## Methods

### Study overview and ethics

**Figure 1:**
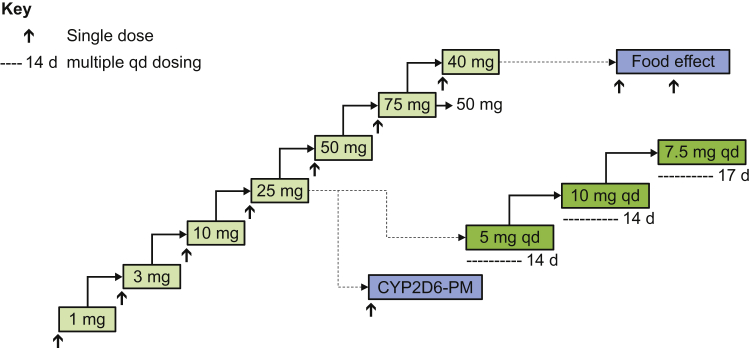
Schematic Overview of the Study Design The study included SAD cohorts, MAD cohorts, a CYP2D6-PM cohort, and a food-effect cohort. The MAD and CYP2D6-PM cohorts began when a tolerated, pharmacologically active dose (reduction in LVEF of approximately 5%) was identified in the SAD cohorts. The food-effect cohort began following completion of the last SAD cohort. Criteria to stop dose escalation were met in the SAD 75-mg dose cohort, and remaining patients in this cohort received 50 mg. Subsequently, the final SAD cohort was completed using 40 mg of aficamten. CYP2D6-PM = cytochrome P450 2D6 poor metabolizer phenotype; d = day; LVEF = left ventricular ejection fraction; MAD = multiple ascending dose; qd = once daily; SAD = single ascending dose.

The study used a randomized, placebo-controlled, single ascending dose (SAD) and multiple ascending dose (MAD) design (Figure 1). The study was not designed to identify a maximum tolerated dose but, rather, to identify a pharmacologically active dose range, defined as giving an absolute reduction in LV ejection fraction (LVEF) from baseline in the range of 5% to 15% (such as a baseline LVEF value of 70% reduced to between 55% and 65%). Dose escalation was to be stopped when this range was achieved or when a nontolerated dose was identified, if earlier.

The study was conducted at a single site (Celerion Clinical Research Unit, Phoenix, Arizona). All study protocols and pertinent study documents were reviewed and approved by the Institutional Review Board (Advarra, Inc, Columbia, Maryland) before study initiation. All participants provided written informed consent. The study was conducted in compliance with the ethical principles set forth in the Declaration of Helsinki and he International Council for Harmonisation TriPartite Guideline regarding Good Clinical Practice and was registered at Clinicaltrials.gov (NCT03767855).

### Participants and treatments

To be eligible for this study, participants were to be healthy adults aged 18 to 55 years, with a body mass index of 18.0 to 32.0 kg/m^2^, and normal electrocardiogram (ECG) and clinical laboratory values or only minor abnormalities that were deemed not clinically significant. Participants also had to have normal cardiac structure and function, with an LVEF of ≥60% for the first 4 SAD cohorts; ≥65% for subsequent SAD cohorts, all MAD cohorts, and the food-effect cohort; and ≥55% for the CYP2D6-PM cohort. With the exception of the CYP2D6-PM cohort, participants were excluded if they were CYP2D6 poor metabolizers based on genotype. Before the study, participants were not allowed to use any prescription medication within 14 days, over-the-counter medication within 7 days (except acetaminophen), or tobacco or nicotine within 3 months; in addition, they were not permitted to consume alcohol, caffeine, or grapefruit within 48 hours before study check-in.

A randomization schedule was centrally generated for each cohort and treatment period. In all cohorts, aficamten or matching placebo was administered in granule form in a capsule with approximately 240 mL of water. Study drug was administered following an overnight fast, except during the fed period in the food-effect cohort.

#### SAD cohorts

The SAD portion of the study used a randomized, double-blind, placebo-controlled, sequential, escalating dose design, in which participants received single ascending oral doses of the study drug. Seven cohorts were dosed in sequence (Figure 1). Of the 8 participants in each cohort, the first 2 were randomly assigned (1:1) to aficamten or placebo and followed up for a minimum of 2 days before the remainder of the group was dosed. The remaining 6 participants were then randomly assigned (5:1) to receive either oral single doses of aficamten (1, 3, 10, 25, 40, 50, or 75 mg) or placebo.

The initial dose of aficamten was selected using criteria from the U.S. Food and Drug Administration guidance,^16^ based on prior animal studies and using a safety margin of ≥10-fold. Dose escalation would stop when results identified a pharmacologically active dose range that reduced LVEF by 5% to 15% or a nontolerated dose, whichever occurred first.

Recommendations regarding dose escalation in the SAD cohorts—and in the MAD cohorts described in the next section—were made by the treating investigator (who was blinded to treatment group) and endorsed or not by the Dose Level Review Committee (DLRC), the members of which were unblinded. Decisions were made when ≥6 participants had been treated and followed up for ≥3 days, including collection of clinical, laboratory, ECG, and telemetry data and echocardiograms suitable for assessing LV function around the time of maximum plasma drug concentration (C_max_). Criteria for escalation included that no more than 2 participants in a dose group developed an LVEF of <50% and that no individual developed an LVEF of <45%; escalation criteria are given in full in the Supplemental Appendix.

#### MAD cohorts

The MAD cohorts also used a randomized, double-blind, placebo-controlled, sequential design. Enrollment in the MAD cohorts began when the SAD cohorts identified a single oral dose that was well tolerated and associated with an observed PD effect. Each of the 3 MAD cohorts included 8 participants, randomized (6:2) to aficamten or placebo. Participants received oral doses of the study drug once daily for 14 days (in the cohorts comparing 5 or 10 mg of aficamten vs placebo) or 17 days (for the cohort comparing 7.5 mg of aficamten vs placebo).

#### CYP2D6-PM cohort

A separate cohort was enrolled to evaluate the potential impact of *CYP2D6* genetic variants on the PK properties of aficamten. The *CYP2D6* gene encodes the cytochrome P450 2D6 enzyme, described as the most extensively characterized polymorphic drug-metabolizing enzyme,^17^ and prior in vitro studies had implicated CYP2D6 as a potential metabolizing enzyme of aficamten.

*CYP2D6* genotypes were determined at screening for all study participants; those identified as CYP2D6-PMs were excluded from the SAD and MAD cohorts but were invited to participate in the CYP2D6-PM cohort. The first individual in the CYP2D6-PM cohort was dosed after the SAD 25-mg cohort (Figure 1). Each participant received a single dose of aficamten (10 mg) or placebo. Nine participants were randomized (7:2), with a sentinel dosing group consisting of the first 2 participants treated.

#### Food-effect cohort

To assess the effect of food on the PK of aficamten, a separate cohort was enrolled after completion of the last SAD cohort, with enrollment of 8 to 12 participants planned. In an open-label, 2-way crossover design, participants were to receive 2 single doses of 10 mg of aficamten, separated by ≥14 days. Participants were randomized in a 1:1 ratio to 1 of 2 sequences: fasted/fed or fed/fasted. In the fasted period, aficamten was administered after an overnight fast; in the fed period, aficamten was administered 30 minutes after the start of a high-fat breakfast.

### Assessments

#### Safety and tolerability

Safety was assessed by the incidence of adverse events (AEs) and by the incidence of reduced LVEF. Treatment-emergent AEs (TEAEs) were defined as AEs that began or increased after study drug administration. All AEs were coded using the Medical Dictionary for Regulatory Activities, version 21.1, and graded using the National Cancer Institute Common Terminology Criteria for AEs (version 4.03) 5-point severity scale.^18^ Each AE was judged as either related or unrelated to the study drug by the treating investigator. Clinical laboratory tests were obtained at regular intervals in all cohorts.

For safety monitoring, participants in all cohorts had periodic echocardiograms, which were assessed by a cardiologist. In the SAD and MAD cohorts, echocardiograms were also reviewed by an echocardiography core laboratory for PD assessments, as described in the section on echocardiographic methods. In addition, participants in all cohorts were monitored with continuous 12-lead ECG recording using Holter monitors. For safety monitoring, a single 12-lead ECG was extracted at screening, predose, and periodically throughout follow-up and interpreted by the investigator. In the SAD, MAD, and CYP2D6-PM cohorts, cardiodynamic ECGs (triplicate 10-second, 12-lead ECG recordings) were obtained before the corresponding PK blood sample (see the Supplemental Appendix for full details on blood sampling), and ECG intervals were quantitated by qualified readers.

#### PK analyses

For all study groups, blood samples for PK assessment were obtained predose, up to 12 times daily on day 1, and then at regular intervals throughout the study (see Supplemental Appendix for details). Standard noncompartmental methods were used to calculate PK parameters using Phoenix WinNonlin, version 7.0; actual sample collection times were used.

Plasma concentrations of aficamten were measured with high-performance liquid chromatography–tandem mass spectrometry methods validated for accuracy, precision, linearity, sensitivity, and specificity at Celerion. The analytic range (the lower to upper limits of quantitation) for aficamten was 1.00 to 500 ng/mL.

#### Echocardiography

For PD assessments of LVEF, echocardiograms for the SAD and MAD cohorts were interpreted by the echocardiography core laboratory and used for all data analysis and dose-level review decisions, and immediate local interpretation of the echocardiograms was performed for safety monitoring. In the SAD cohorts receiving 1, 3, or 10 mg of aficamten, echocardiograms were obtained on day -1; predose on day 1; and at 1.5, 4, and 24 hours postdose. For SAD cohorts receiving 25, 40, 50, or 75 mg of aficamten, echocardiograms were obtained on day -1; predose on day 1; and at 1.5, 6, and 24 hours postdose. Echocardiograms on day 3 (48 hours after dosing) were obtained only if the 24-hour LVEF had not returned to near or above baseline, as determined by the investigator. In the MAD cohorts, echocardiograms were obtained on day -1; predose on day 1; at 1.5 hours postdose on days 2, 4, and 9; and at 1.5, 24, and 72 hours postdose on day 14 (for the 5-mg and 10-mg cohorts) or on day 17 (for the 7.5-mg cohort). Echocardiograms were obtained 3 days after the last dose (on day 17 or 20) only if the participant’s prior LVEF was not near or above baseline, as determined by the investigator.

Using echocardiography instrumentation certified for clinical use, echocardiographic images were obtained from standard views based on American Society of Echocardiography recommendations for chamber quantification.^19^ To obtain the LVEF, the endocardial borders of 3 different cardiac cycles were traced at both end diastole and end systole from the apical 4- and 2-chamber views and LV end-diastolic volume (LVEDV) and LV end-systolic volume (LVESV), calculated using the Simpson’s method of discs algorithm. Ejection fraction was calculated in the standard manner: LVEF = (LVEDV − LVESV)/LVEDV. The intraobserver variability (coefficient of variation) for LVEF was 8%.

### Statistical analysis

The sample size chosen for this study was based on precedent set by other first-in-human PK studies of a similar nature and was not based on power calculations. All participants who received ≥1 dose of the study drug (aficamten or placebo) were included in safety analyses. All participants who received ≥1 dose of the study drug and had ≥1 evaluable PK plasma profile were included in the PK analysis set. Dose-proportionality analysis and steady-state analyses are described in the Supplemental Appendix.

All participants who received ≥1 dose of the study drug and had ≥1 predose and ≥1 postdose echocardiographic measurements were included in the PD analysis set. Descriptive analyses included absolute reduction in LVEF relative to baseline and categorical LVEF responses (proportions of participants with reduction in LVEF from baseline of ≥5%, ≥10%, and >15% and proportions of participants with LVEF of <50% and <45%). Descriptive statistics of the echocardiographic parameters were generated using SAS, version 9.3 or higher. Dose-response analysis was performed using analysis of covariance to identify the least-squares mean difference (aficamten minus placebo), as described in the Supplemental Appendix. Analyses of concentration “bin” and exposure-response were also performed using analysis of covariance, as described in the Supplemental Appendix. SAS PROC MIXED was used for all comparative analyses.

All timepoints for which both PK data and PD measures were available were pooled for the analysis. A nominal significance level of 5% was used for statistical comparisons, without adjustment for multiplicity.

## Results

### Study population

The study was conducted from November 8, 2018, through January 3, 2020. A total of 102 participants were enrolled: 57 in the SAD cohorts, 24 in the MAD cohorts, 9 in the CYP2D6-PM cohort, and 12 in the food-effect cohort. All participants completed the study. Mean age ranged between 32 and 40 years across cohorts, and the majority of participants were men (Table 1).

**Table 1:** Baseline Characteristics

	SAD (n = 57)	MAD (n = 24)	CYP2D6-PM (n = 9)	Food Effect (n = 12)
Age, y	39.6 (18-55)	40.4 (28-54)	32.0 (20-47)	39.6 (25-51)
Male	41 (72)	18 (75)	9 (100)	9 (75)
White race	52 (91)	17 (71)	9 (100)	11 (92)
Hispanic or Latino ethnicity	42 (74)	13 (54)	4 (44)	8 (67)
Weight, kg	79.4 ± 10.3	77.0 ± 9.1	84.8 ± 12.0	76.4 ± 11.1
Height, cm	169.0 ± 9.4	168.9 ± 10.0	177.8 ± 7.8	168.8 ± 9.2
BMI, kg/m^2^	27.8 ± 2.7	27.0 ± 2.3	26.8 ± 3.3	26.8 ± 3.0
LVEF, %	65.8 ± 2.4	67.5 ± 1.2	61.0 ± 2.6	67.2 ± 1.2

Values are mean (range), n (%), or mean ± SD.

BMI = body mass index; CYP2D6-PM = cytochrome P450 2D6 poor metabolizer; LVEF = left ventricular ejection fraction; MAD = multiple ascending dose; SAD = single ascending dose.

For the SAD cohorts, there were no safety concerns that prohibited dose escalation between 1 mg and 25 mg. With the next planned dose (50 mg), 1 participant had a postdose LVEF of <50% (46.2%); however, this did not meet the dose escalation stopping rules, and the 75-mg cohort was initiated. The sentinel participant in the 75-mg cohort had an LVEF of <45% postdose; consequently, no further participants were dosed at 75 mg. As a result, the 50-mg group was expanded, and an additional 5 participants were dosed in this cohort. Following the expansion, 1 participant in the 50-mg dose group experienced an LVEF of <45%, which was also a decrease of >15%. Therefore, no further participants were dosed at ≥50 mg. Given that the 50-mg dose was well tolerated by all participants, the investigator recommended a dose of 40 mg for the final single-dose cohort, with the rationale that a dose between 25 mg and 50 mg may add value to determining the effect of aficamten on LVEF. The DLRC concurred and endorsed this recommendation.

Following results from the 1-mg to 25-mg SAD cohorts, the first MAD cohort was initiated at 5 mg of aficamten once daily for 14 days. There were no safety concerns, and the next cohort was initiated at 10 mg once daily for 14 days. In this cohort, 2 participants met the stopping criteria based on echocardiography results. The DLRC decided the next treatment level should be 7.5 mg to better characterize the PK at the steady state; thus, the dosing period was extended from 14 to 17 days to ensure the PK had reached a steady state by the last day of dosing.

### Safety and tolerability

There were no serious AEs, and no participants discontinued the study because of AEs. The TEAEs that were observed were generally mild (grade 1) and no more frequent with aficamten than with placebo for both single-dose and multiple-dose administration (Tables 2 and 3). Overall, the most common TEAE was headache in both the SAD and MAD cohorts (Tables 2 and 3).

**Table 2:** TEAEs in the SAD Cohorts

	Pooled Placebo (n = 15)	Aficamten SAD Cohort, mg							Total (N = 57)
		1 (n = 6)	3 (n = 6)	10 (n = 6)	25 (n = 6)	40 (n = 6)	50 (n = 11)	75 (n = 1)	
Participants with any TEAE	4 (27)	1 (17)	—	1 (17)	5 (83)	1 (17)	4 (36)	1 (100)	17 (30)
Most common TEAEs									
Headache	1 (7)	—	—	1 (17)	2 (33)	—	1 (9)	—	5 (9)
Ejection fraction decreased	—	—	—	—	—	1 (17)	1 (9)	1 (100)	3 (5)
Abdominal tenderness	—	—	—	—	1 (17)	—	1 (9)	—	2 (4)
Chest pain	—	—	—	—	—	—	1 (9)^a^	1 (100)^b^	2 (4)
Dyspepsia	—	—	—	—	1 (17)	—	1 (9)	—	2 (4)
Flatulence	—	—	—	—	1 (17)	—	1 (9)	—	2 (4)
Nausea	—	—	—	—	2 (33)	—	—	—	2 (4)
Upper abdominal pain	—	—	—	—	2 (33)	—	—	—	2 (4)
Upper respiratory tract infection	2 (13)	—	—	—	—	—	—	—	2 (4)

Values are n (%). The most common TEAEs are based on preferred terms, reported in ≥2 participants in the total cohort.

SAD = single ascending dose; TEAE = treatment-emergent adverse event.

a “Bubbling sensation to the left chest” consistent with a gastrointestinal association rather than a cardiac association.

b Onset approximately 60 hours postdose.

**Table 3:** TEAEs in the MAD Cohorts

		Aficamten MAD Cohorts			
	Pooled Placebo (n = 6)	5 mg qd × 14 d (n = 6)	7.5 mg qd × 17 d (n = 6)	10 mg qd × 14 d (n = 6)	Total (N = 24)
Participants with any TEAE	1 (17)	1 (17)	—	2 (33)	4 (17)
TEAEs					
Headache	1 (17)	—	—	1 (17)	2 (8)
Chapped lips	—	1 (17)	—	—	1 (4)
Cough	—	1 (17)	—	—	1 (4)
Feeling hot	1 (17)	—	—	—	1 (4)
Nausea	1 (17)	—	—	—	1 (4)
Salivary hypersecretion	—	1 (17)	—	—	1 (4)
Upper respiratory tract infection	—	—	—	1 (17)	1 (4)
Vomiting	1 (17)	—	—	—	1 (4)

Values are n (%). TEAEs are based on preferred terms, reported in ≥1 participant in the total cohort.

MAD = multiple ascending dose; qd = once daily; TEAE = treatment-emergent adverse event.

Echocardiogram-related AEs of decreased LVEF of <45% based on the study echocardiogram expert assessment were reported in 3 participants: 1 each in the SAD 40-mg, 50-mg, and 75-mg cohorts (Supplemental Table 1). All were grade 1, and all resolved at the next echocardiogram assessment (within 2.5-4.6 hours). The single participant who received 75 mg of aficamten had an LVEF of 34.6% at 1.5 hours postdose, which was a reduction in LVEF of 31.5% and led to concluding escalation of doses in the SAD portion of the study, as discussed earlier. At the following assessment 2.5 hours later, the LVEF had returned to 51.9%. No AEs of decreased ejection fraction of <45% were reported in the MAD, CYP2D6-PM, or food-effect cohorts.

In all cohorts, mean safety ECG parameters at the assessed timepoints were within normal limits. No clinically notable changes from baseline were observed among any of the parameters. The QT interval corrected for heart rate using Fridericia’s formula (QTcF) did not exceed 450 ms either at baseline or at any assessment during the dosing interval, with the exception of 2 individuals whose baseline QTcF was ≥440 ms and whose QTcF values increased by 3 and 13 ms, respectively. In all cohorts, there were no increases in QTcF interval of >30 ms, with the exception of 1 participant in the SAD placebo group whose QTcF interval increased by 33 ms on day 5 (427 ms vs a baseline value of 394 ms). In the cardiodynamic assessments, categorical analysis of ECG parameters revealed no cardiac safety concerns, and there was no evidence of an effect on the QT interval following single or multiple doses of aficamten.

All vital signs were within normal limits at the postdose timepoints. No clinically significant serum chemistry, hematology, or urinalysis findings were observed during the study.

### Pharmacokinetics

#### Single-dose kinetics

**Figure 2:**
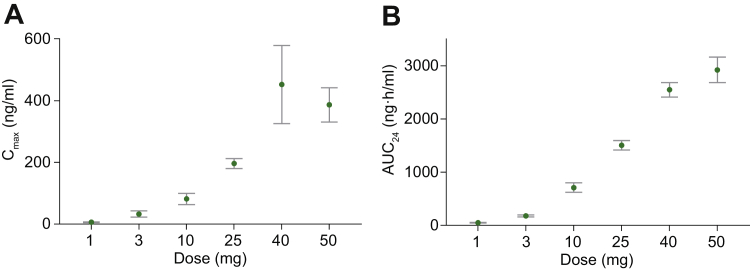
Maximum Plasma Concentrations and Exposure With Single Doses of Aficamten **(A)** Mean (SE) C_max_ and **(B)** exposure (AUC_24_) of aficamten increased in a dose-proportional manner following single oral doses between 1 mg and 50 mg. AUC_24_ = area under the plasma drug concentration–time curve from 0 to 24 hours; C_max_ = maximum plasma concentration.

The plasma aficamten profiles were generally well characterized for all dose levels with the exception of the lowest dose of 1 mg (because of concentrations close to the lower limit of quantitation) and the highest dose of 75 mg, which was administered to only 1 participant, as discussed earlier. Aficamten plasma concentration over time is shown in Supplemental Figure 1. Over the dose range of 1 to 50 mg, mean maximal plasma concentrations and exposure increased in a dose-proportional manner, as demonstrated by the rise in C_max_ and area under the plasma concentration–time curve from time 0 to 24 hours (AUC_24_) with increasing doses (Figure 2, Table 4). Mean clearance and volume of distribution were similar across the doses. Median time to maximum observed concentration occurred between 0.5 and 2.8 hours, with a maximum time of 4.0 hours across all participants. Mean half-life ranged from 75 to 85 hours.

**Table 4:** Summary of Plasma Aficamten Pharmacokinetics Following Single Oral Dose Administration

	Aficamten SAD Cohort, mg						
	1	3	10	25	40	50	75
C_max_, ng/mL							
	5.8 (38.4)	26.1 (82.4)	70.5 (69.0)	192.6 (23.3)	383.2 (67.9)	359.4 (38.9)	1,220 (-)
	n = 6	n = 6	n = 6	n = 6	n = 6	n = 11	n = 1
T_max_, h							
	1.0 (0.5-4.0)	1.3 (0.5-2.6)	2.8 (0.5-3.0)	1.8 (0.5-4.0)	1.0 (0.5-1.5)	1.0 (0.5-2.5)	0.5 (0.5-0.5)
	n = 6	n = 6	n = 6	n = 6	n = 6	n =11	n = 1
AUC_24_, ng·h/mL							
	50 (20)	174 (27)	679 (35)	1,493 (15)	2,532 (13)	2,833 (26)	4,556 (—)
	n = 6	n = 6	n = 6	n = 6	n = 6	n = 11	n = 1
AUC_inf_, ng·h/mL							
	—	823 (61)	3,434 (24)	7,113 (15)	11,860 (20)	13,740 (35)	
	—	n = 5	n = 4	n = 6	n = 5	n = 9	
Vz/F, L							
	—	392.7 ± 122.2	361.7 ± 108.0	405.3 ± 56.6	381.7 ± 66.9	431.3 ± 122.9	
	—	n = 5	n = 4	n = 6	n = 5	n = 9	
t_1/2_, h							
	—	75.1 ± 21.8	84.7 ± 17.7	79.8 ± 10.0	79.2 ± 18.5	80.4 ± 14.9	
	—	n = 5	n = 4	n = 6	n = 4	n = 9	
CL/F, L/h							
	—	4.2 ± 3.1	3.0 ± 0.7	3.6 ± 0.5	3.4 ± 0.6	3.8 ± 1.1	
	—	n = 5	n = 4	n = 6	n = 5	n = 9	

Values are geometric mean (geometric CV%), median (range), or mean ± SD.

AUC_24_ = area under the plasma drug concentration–time curve from time 0 to 24 hours; AUC_inf_ = area under the plasma drug concentration–time curve from time 0 extrapolated to infinity; CL/F = apparent total body clearance; C_max_ = maximum plasma concentration; CV% = percent coefficient of variation; t_1/2_ = half-life; T_max_ = time to maximum plasma concentration; Vz/F = apparent volume of distribution.

#### Multiple-dose kinetics

**Figure 3:**
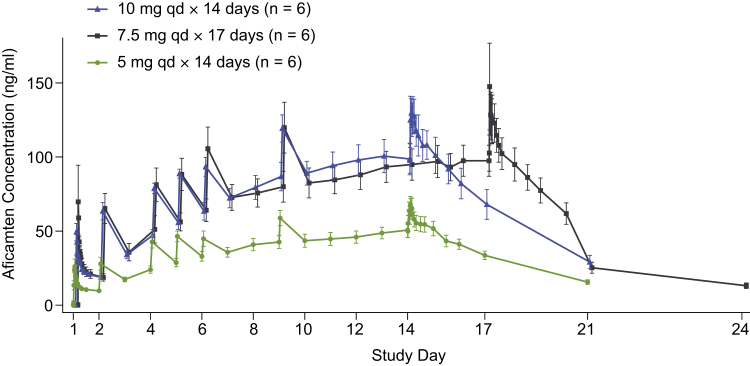
Plasma Concentration Over Time With Multiple Doses of Aficamten Mean (SE) aficamten plasma concentrations are displayed. Data points are offset for clarity. Aficamten plasma concentrations increased between the 5-mg dose and the 2 higher doses; however, by day 2, there was no difference between mean concentrations of the 7.5-mg and 10-mg doses. Clearance was similar for the 5-mg and 10-mg doses, and the accumulation ratio was similar for all 3 doses. For days 7, 8, 10, 11, 12, and 13, only trough measurements are shown. For the 5-mg and 10-mg cohorts, the dosing period was 14 days with a 3-day follow-up. For the 7.5-mg cohort, dosing was extended to 17 days with a 3-day follow-up, and it was confirmed that the steady state was achieved after 10 to 12 days. qd = once daily.

With once-daily dosing, mean plasma concentrations increased between the 5-mg dose and the 2 higher doses (7.5 mg and 10 mg); however, by day 2, there was little difference between mean concentrations of the 7.5-mg and 10-mg doses (Figure 3). Plasma PK parameters are displayed in Table 5. By the end of the treatment period (day 14 or 17), the mean plasma concentration was between 2 and 2.5 times that of day 1. Terminal elimination half-life estimates were consistent across doses, ranging between 77 and 86 hours. Clearance was similar for the 5- and 10-mg doses, and the accumulation ratio was similar for the 3 doses. Consistent with the observed terminal elimination half-life estimates, a steady state was achieved after 10 to 12 days (Figure 3).

**Table 5:** Summary of Plasma Aficamten Pharmacokinetics Following Multiple Oral Dose Administration

	MAD Cohorts		
	5 mg qd × 14 d (n = 6)	7.5 mg qd × 17 d (n = 6)	10 mg qd × 14 d (n = 6)
Day 1			
C_max_, ng/mL	28.0 (30.1)	69.3 (66.5)	54.4 (25.1)
T_max_, h	1.3 (0.5-2.5)	0.8 (0.5-1.5)	1.0 (1.0-2.5)
AUC_24_, ng·h/mL	278 (21)	551 (33)	547 (11)
Day 14/17			
C_max,ss_, ng/mL	69.0 (23)	147.6 (39.5)	141.2 (19.7)
T_max,ss_, h	2.8 (1.5-4.0)	1.0 (0.5-5.0)	2.5 (0.5-3.0)
AUC_tau_, ng·h/mL	1,320 (23)	2,518 (26)	2,632 (23)
t_½_, h	86.4 ± 11.9	76.9 ± 14.5	79.8 ± 14.1
CL_ss_/F, h	3.9 ± 0.9	3.1 ± 0.7	3.9 ± 0.8
RA_,AUC_	4.8 ± 0.2	4.6 ± 0.7	4.9 ± 0.9

Values are geometric mean (geometric CV%), median (range), or arithmetic mean ± SD.

AUC_24_ = area under the plasma drug concentration–time curve from time 0 to 24 h; AUC_tau_ = area under the curve to the end of the dosing period; CL_ss_/F = apparent total body clearance after oral administration (at steady state); C_max_ = maximum plasma concentration; C_max,ss_ = maximum plasma concentration at steady state; CV% = percent coefficient of variation; MAD = multiple ascending dose; qd = once daily; RA,_AUC_ = accumulation ratio calculated from AUC_tau_ at the steady state and following a single dose; t_1/2_ = half-life; T_max_ = time to maximum plasma concentration; T_max,ss_ = time to reach maximum plasma concentration following drug administration at steady state.

#### CYP2D6-PM cohort

The PK parameters of aficamten in the CYP2D6-PM cohort are displayed in Supplemental Figure 2 and Supplemental Table 2. In the CYP2D6-PM group, the mean half-life was prolonged to 110 hours, compared with 85 hours in extensive metabolizers (ie, the 10-mg SAD cohort); however, no increase in the AUC was observed in this group, with a geometric mean AUC_24_ of 495 ng·h/mL (geometric percent coefficient of variation [CV%]: 19) (Supplemental Table 2), compared with 679 ng·h/mL (geometric CV%: 35) in extensive metabolizers (Table 4). The CYP2D6-PM group did not appear to have reduced clearance that resulted in a clinically meaningful difference in exposures.

#### Effect of food

The PK parameters of aficamten in the food-effect cohort are displayed in Supplemental Figure 3 and Supplemental Table 3. When taken with food, the C_max_ of aficamten increased by approximately 30%, and the time to maximum observed concentration was reduced (1.5 vs 2.3 hours). However, food had little effect on the AUC, with a geometric mean AUC_24_ in the fasted state of 601 ng·h/mL (geometric CV%: 33) vs 631 ng·h/mL (geometric CV%: 25) in the fed state.

### Pharmacodynamics

#### Echocardiography

**Figure 4:**
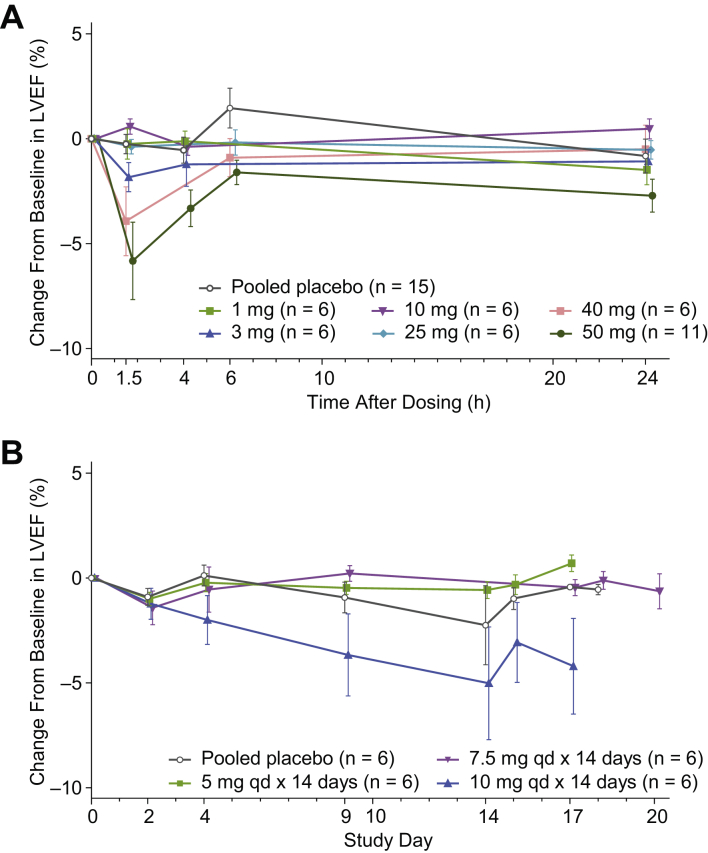
Change From Baseline in LVEF **(A)** SAD cohorts. **(B)** MAD cohorts. Mean (SE) change from baseline in LVEF is displayed. Data points are offset for clarity. In both the SAD and MAD cohorts, reductions in LVEF within the target range (5%-15% reduction) were observed. In the SAD cohorts, there were generally small decreases in LVEF, with mean maximum reduction of 5.8% in the 50-mg group (at 1.5 hours postdose). In the MAD cohort, the greatest mean reduction in LVEF from baseline occurred in the 10-mg group (mean change of 5.0% 1.5 hours after dosing on day 14). Abbreviations as in Figure 1.

At baseline, mean LVEF ranged from 61.0% to 67.5% across cohorts (Table 1). In the SAD cohorts, mean decreases in LVEF were observed in the groups receiving the highest doses of aficamten (Figure 4A). Maximum mean reduction from baseline in LVEF was seen in the 50-mg cohort at 1.5 hours postdose (placebo corrected least- squares mean difference: 5.5%; *P* < 0.001). LVESV and LVEDV were statistically significantly increased by 8.1 and 6.6 mL, respectively (Supplemental Table 4). Other echocardiographic parameters such as stroke volume, cardiac output, cardiac time intervals, and measures reflective of diastolic function did not significantly change (Supplemental Table 4). The single participant who received 75 mg of aficamten exhibited reduction in LVEF of 31.5% at 1.5 hours postdose, which resolved 2.5 hours after onset but led to concluding escalation of doses in the SAD portion of the study, as discussed earlier.

In the MAD cohorts, a clear decrease in LVEF emerged as dosing continued in the 10-mg cohort (Figure 4B). The largest mean maximum percent reduction from baseline, of 5.0%, was seen in the 10-mg cohort at 1.5 hours postdose on day 14 (Figure 4B). The placebo-corrected reduction of 3.2% (least-squares mean difference) did not reach statistical significance (*P* = 0.21), likely because of the lack of statistical power in this small group comparison.

#### Categorical LVEF responses

In the SAD cohorts, absolute reductions in LVEF of ≥5% from baseline were observed in 1 of 15 (7%) participants in the placebo cohort, 1 of 6 (17%) in the 3-mg cohort, 2 of 6 (33%) in the 40-mg cohort, 7 of 11 (64%) in the 50-mg cohort, and 1 of 1 (100%) in the 75-mg cohort, whereas no participants in the 1-, 10-, or 25-mg cohorts had a reduction of ≥5%. Absolute reductions in LVEF of ≥10% occurred in 1 of 6 (17%) participants in the 40-mg cohort, 2 of 11 (18%) in the 50-mg cohort, and 1 (100%) in the 75-mg cohort. Reduction in LVEF to <50% was observed for 2 of 11 (18%) participants in the 50-mg cohort (48.2% and 45.5% per core laboratory assessment) and 1 of 1 (100%) in the 75-mg cohort. Only the participant in the 75-mg cohort experienced an LVEF of <45%.

In the MAD cohort, absolute reduction in LVEF of ≥5% from baseline was observed in 4 participants: 1 of 6 (17%) participants receiving placebo, 1 of 6 (17%) receiving 7.5 mg of aficamten once daily, and 2 of 6 (33%) receiving 10 mg of aficamten once daily. Of these, reductions were ≥10% in the 2 participants in the 10-mg cohort. Reduction in LVEF to <50% was not observed in any of the MAD cohorts per core laboratory assessment.

### Relationship of plasma concentration to change in LVEF

**Figure 5:**
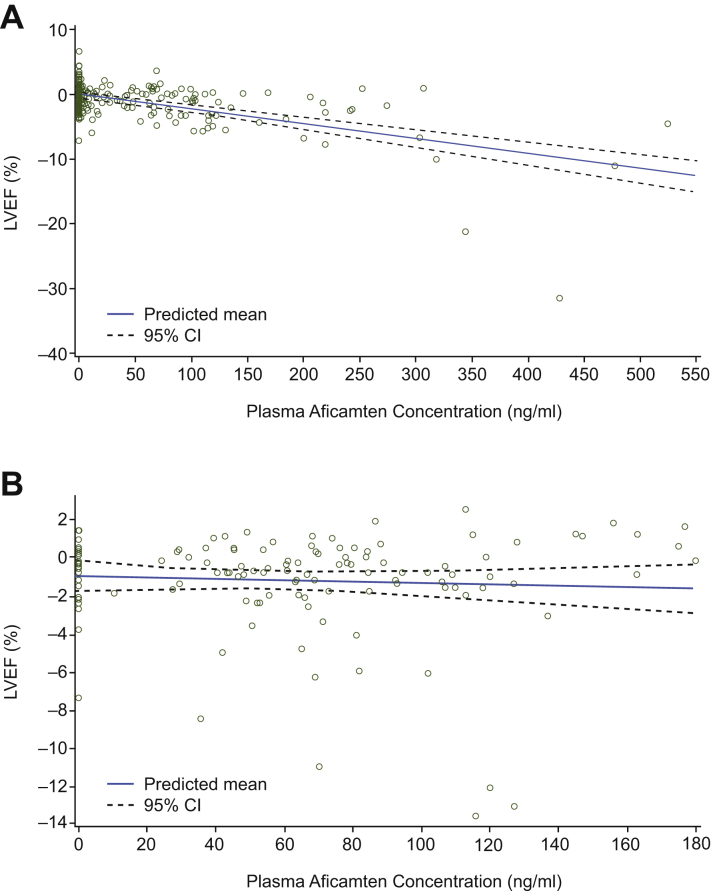
Change From Baseline in LVEF vs Time-Matched Plasma Concentration of Aficamten **(A)** Analysis of the SAD cohorts showed that as the plasma concentration of aficamten increased, there was a trend toward a decrease in LVEF. **(B)** Analysis of the MAD cohorts showed minimal suppression of LVEF in most participants at plasma aficamten concentrations of ≤180 ng/mL. Abbreviations as in Figure 1.

The PK/PD relationship for aficamten is illustrated by plotting the plasma concentration of aficamten vs change in LVEF for the SAD and MAD cohorts (Figure 5). In the SAD cohort, as plasma concentration of aficamten increased, there was a trend toward a decrease in LVEF. The relationship of LVEF to the plasma concentration of aficamten was statistically significant, both in the bin concentration analysis for the highest plasma concentration bin (122-524 ng/mL; *P <* 0.001) and in the concentration-slope analysis (*P* = 0.003). In the MAD cohorts, the relationship of LVEF to plasma aficamten did not reach statistical significance in the bin concentration analysis or the linear regression analysis, likely because of the more limited range of plasma concentrations explored and the small group sizes.

## Discussion

This phase 1, first-in-human study has established the doses (up to 50 mg as a single oral dose or up to 10 mg following multiple doses) at which aficamten was both physiologically effective at reducing LVEF and was well tolerated in healthy participants, identifying pharmacologically active doses that will serve as starting doses for a study in patients with HCM. In addition, single oral doses of 10 mg were well tolerated among individuals with the CYP2D6-PM phenotype, and there was no significant effect of food on the PK of aficamten. Collectively, these observations support the continued development of aficamten for patients with HCM and provide a roadmap for phase 2 studies.

### Safety of aficamten

We observed no serious AEs in the study, and all participants completed the intended dosing as planned. Generally, AEs were mild and similar in frequency between participants treated with aficamten and placebo. Importantly, there were no associated symptoms or adverse changes in vital signs for participants whose LVEFs fell below 50%, and the LVEFs in these individuals returned to baseline within 24 hours. This study was not intended to find a maximum tolerated dose, and hence, dose escalation stopped once a clear PD effect was observed in the SAD and MAD portions of the study; thus, a dose that was not tolerated because of AEs was not identified.

### Effect on LVEF

In the SAD cohorts, a dose of 50 mg produced a placebo-corrected least-squares mean reduction in LVEF of 5.5%, and in the MAD cohort, 10 mg once daily for 14 days produced a placebo-corrected least-squares mean reduction in LVEF of 3.2%. The proportion of participants with absolute reductions in LVEF of ≥5% from baseline increased as the dose increased; up to 64% of participants in the 50-mg SAD cohort and 33% in the 10-mg MAD cohort had absolute reductions in LVEF of ≥10% from baseline. In the SAD cohorts, where the broadest range of exposures of aficamten was explored, there was a statistically significant decrease in LVEF as plasma concentrations of aficamten increased. Thus, the study achieved its secondary objective of identifying a pharmacologically active dose and describing its PK/PD relationship.

Three participants had decreases in LVEF to <50% that were rapidly reversible upon study drug discontinuation. Following a single dose of 50 mg, 2 (18%) participants experienced an LVEF of <50% (48.2% and 45.5%). After a single dose of 75 mg, 1 participant experienced reduction of LVEF to 34.1%. In all cases, the event was noted approximately 1.5 hours after dosing, and LVEF recovered to >50% by 4 to 6 hours after dosing. The SAD results informed dose selection for the other portions of the study, and there were no echocardiographic AEs in the MAD, CYP2D6-PM, or food-effect cohorts.

### Implications of PK results

Aficamten demonstrated linear kinetics over the dose range of 1 mg to 50 mg; half-life was independent of concentration, and clearance was independent of dose. Steady-state was achieved by the end of day 10 with the 10-mg dose and by the end of day 12 with the 5-mg and 7.5-mg doses. There was no effect of food suggestive of a need to alter dosing. These findings support once-daily dosing in either the fasted or fed state.

The relationship between plasma concentration and LVEF suggests a broad therapeutic index, which will facilitate optimization of individual doses in patients with HCM, who are expected to be titrated through an escalating range of doses until the desired PD effect is achieved. In addition, the half-life of aficamten (75-85 hours following a single dose and 77-86 hours following multiple doses) and observed reversibility of effect offers a potential advantage in that steady state is achieved within 2 weeks, and excessive effects on LVEF are readily reversed.

### Study limitations

Strengths of the study include the double-blind, randomized, placebo-controlled treatment groups as well as the frequency of laboratory evaluations and echocardiograms. However, the number of participants at each dose was not large, which may have limited statistical analyses. In addition, as with all studies in healthy participants, the PK/PD findings should be extrapolated with caution to patients receiving concomitant medications that may have their own negative inotropic effects, as is typical of patients with HCM.

## Conclusions

Aficamten demonstrated a favorable safety profile in healthy participants, without serious AEs or meaningful changes in laboratory tests, ECGs, or health assessments. The PK in humans supports once-daily dosing and attainment of the steady state within 2 weeks. There was no effect of food or CYP2D6 phenotype on the PK of aficamten. Pharmacologically active doses of aficamten that may serve as starting doses for a study in patients with HCM were identified. Any decreases in LVEF to values of <50% were reversible within 6 hours following single doses*.* In sum, these phase 1 data support the conclusion that the preclinical profile of aficamten translates into humans and warrant further clinical evaluation of aficamten. In that regard, a phase 2 trial (REDWOOD-HCM; NCT04219826) to understand the effect of different doses of aficamten in patients with HCM^20^ has yielded data supporting the progression of aficamten into a phase 3 trial (SEQUOIA-HCM; NCT05186818), which is underway.^21^

Perspectives **COMPETENCY IN MEDICAL KNOWLEDGE:** Sarcomere dysregulation has been proposed as the pathophysiologic basis for certain genetic HCMs. Through inhibition of the cardiac myosin ATPase, cardiac myosin inhibitors reduce the number of active force generators in the sarcomere and have the potential to selectively target the hypercontractile state of the sarcomere in HCM. By addressing the sarcomeric basis for the disease, cardiac myosin inhibitors such as aficamten may slow or reverse the progression of myocyte disarray, interstitial fibrosis, and cardiac hypertrophy associated with HCM.

**TRANSLATIONAL OUTLOOK:** Prior in vitro and in vivo studies have demonstrated that aficamten reduces myocardial contractility. This first-in-human, phase 1 study of aficamten demonstrated a statistically significant reduction in LVEF and established the dosing parameters for aficamten for use in future studies of patients with HCM. Aficamten represents a potential treatment option for HCM, and these data support further clinical investigations of aficamten.

## Funding Support and Author Disclosures

The study was funded by Cytokinetics, Inc. At the time of the study, Dr Malik, Dr Robertson, Mr Robbie, Dr Osmukhina, and Ms Xu, were employed by and owned stock in Cytokinetics, Inc. Dr Armas was employed by Celerion, Inc. Dr Li was employed at Certara, Inc. Drs Li and Solomon are consultants to Cytokinetics, Inc. Dr Solomon has received research support from Cytokinetics, Inc; has received research grants from Actelion, Alnylam, Amgen, AstraZeneca, Bellerophon, Bayer, Bristol Myers Squibb, Celladon, Cytokinetics, Eidos, Gilead, GlaxoSmithKline, Ionis, Lilly, Mesoblast, MyoKardia, National Institutes of Health/National Heart, Lung, and Blood Institute, Neurotronik, Novartis, Novo Nordisk, Respicardia, Sanofi Pasteur, Theracos, and US2.AI; and has consulted for Abbott, Action, Akros, Alnylam, Amgen, Arena, AstraZeneca, Bayer, Boehringer Ingelheim, Bristol Myers Squibb, Cardior, Cardurion, Corvia, Cytokinetics, Daiichi-Sankyo, GlaxoSmithKline, Lilly, Merck, MyoKardia, Novartis, Roche, Theracos, Quantum Genomics, Cardurion, Janssen, Cardiac Dimensions, Tenaya, Sanofi-Pasteur, Dinaqor, Tremeau, CellProThera, Moderna, American Regent, and Sarepta. All other authors have reported that they have no relationships relevant to the contents of this paper to disclose.
