# Supplementary material for: A Phase 1 Dose-Escalation Study of the Cardiac Myosin Inhibitor Aficamten in Healthy Participants
Source: JACC Basic Transl Sci. 2022 Aug 10;7(8):763–75. doi: 10.1016/j.jacbts.2022.04.008 (PMC9436819; doi:10.1016/j.jacbts.2022.04.008)
Supplement: Supplemental Data [file mmc1.pdf]

**SUPPLEMENTAL APPENDIX**


| Supplemental Figures and Tables | Page |
|---|---|
| Dose Escalation Criteria | 2 |
| Blood Sample Collection | 3 |
| Statistical Analysis of Pharmacokinetic (PK) Parameters | 4 |
| Statistical Analysis of Pharmacodynamic (PD) Parameters | 6 |
| SUPPLEMENTAL TABLE 1: Echocardiogram-Related AEs of Decreased Ejection Fraction <45% | 8 |
| SUPPLEMENTAL TABLE 2: Summary of Plasma Aficamten Pharmacokinetics in the CYP2D6 Poor Metabolizer Cohort | 9 |
| SUPPLEMENTAL TABLE 3: Summary of Plasma Aficamten Pharmacokinetics in the Food-Effect Cohort | 10 |
| SUPPLEMENTAL TABLE 4: Echocardiographic Parameters | 11 |
| SUPPLEMENTAL FIGURE 1: Aficamten Plasma Concentration Over Time With Single Doses of Aficamten | 12 |
| SUPPLEMENTAL FIGURE 2: Plasma Aficamten Concentration Over Time in CYP2D6 Poor Metabolizers | 13 |
| SUPPLEMENTAL FIGURE 3: Plasma Aficamten Concentration Over Time Profiles in Fed or Fasted Conditions | 14 |

## ADDITIONAL METHODOLOGY

### Dose Escalation Criteria

- No individual had sustained a cardiac serious adverse event related to the study drug.
- No 2 individuals had experienced similar, non-cardiac serious adverse events in the same organ system that seemed to be related to the study drug.
- No 2 individuals treated with aficamten experienced a decrease in left ventricular ejection fraction (LVEF) >15% in comparison with the last pre-dose value (determined by the Dose Level Review Committee [DLRC]).
- No more than 2 individuals developed an LVEF <50% (unless determined not to be related to the study drug by the DLRC and the treating investigator).
- No individual developed an LVEF <45% (unless determined not to be related to the study drug by the DLRC and the treating investigator).
- Both the treating investigator and DLRC approved the escalation and next level dose based on their clinical judgment.

**Blood Sample Collection**

Blood samples were collected according to the schedule outlined below:

| | |
|---|---|
| SAD Cohorts | Day 1: pre-dose and 0.25, 0.5, 1, 1.5, 2, 2.5, 3, 4, 6, 8, 12, 16, 24, 36, 48, 72, 96, and 216 h post dose |
| MAD Cohorts (14-Day Dosing) | Day 1: pre-dose and 0.25, 0.5, 1, 1.5, 2, 2.5, 3, 4, 6, 8, and 12 h post dose |
| | Days 2, 4, 5, 6, and 9: pre-dose (corresponding to trough samples following dosing on days 1, 3, 4, 5, and 8) and 1.5 h post dose |
| | Days 3, 7, 8, 10, 11, 12, and 13: pre-dose (corresponding to trough samples following dosing on days 2, 6, 7, 9, 10, 11, and 12) |
| | Day 14: pre-dose and 0.25, 0.5, 1.5, 2, 2.5, 3, 4, 6, 8, 12, 16, 24, 36, 48, 72, and 168 h post dose |
| MAD Cohort (17-Day Dosing) | Day 1: pre-dose and 0.25, 0.5, 1, 1.5, 2, 2.5, 3, 4, 6, 8, and 12 h post dose |
| | Days 2, 4, 5, 6, and 9: pre-dose (corresponding to trough samples following dosing on days 1, 3, 4, 5, and 8) and 1.5 h post dose |
| | Days 3, 7, 8, 10, 11, 12, 13, 14, 15, and 16: pre-dose (corresponding to trough samples following dosing on days 2, 6, 7, 9, 10, 11, and 12) |
| | Day 17: pre-dose and 0.25, 0.5, 1.5, 2, 2.5, 3, 5, 7, 9, 12, 24, 36, 48, 72, and 168 h post dose |
| CYP2D6-PM Cohort | Day 1: pre-dose and 0.25, 0.5, 1, 1.5, 2, 2.5, 3, 4, 6, 8, 12, 16, 24, 36, 48, 72, 96, 216, 312, and 552 h post dose |
| Food-Effect Cohort | Day 1: pre-dose and 0.25, 0.5, 1, 1.5, 2, 2.5, 3, 4, 6, 8, 12, 16, 24, 36, 48, 72, 96, 144, and 216 h post dose |

**Statistical Analysis of Pharmacokinetic (PK) Parameters**

The PK analyses were intended to assess single-dose kinetics, multiple-dose (steady state) kinetics, the influence of the CYP2D6 phenotype on absorption and elimination of aficamten, and the influence of food on the absorption and elimination of aficamten.

*Dose proportionality analysis*

For the SAD cohort, dose proportionality of aficamten was evaluated using a power model on day 1. For the MAD cohort, dose proportionality was evaluated using a power model on day 1 and day 14 or 17. Several considerations were taken into account when assessing dose proportionality of the drug, such as results derived from the power model statistical analysis (e.g., the slope estimate and width of the 2-sided 95% confidence intervals [CIs]), qualitative assessment specific to the PK of the drug, and clinical relevance. For the SAD cohort, the parameters used to assess dose proportionality were area under the plasma drug concentration–time curve (AUC) from time 0 to the time of the last measurable concentration ($AUC_{last}$), AUC from time 0 extrapolated to infinity ($AUC_{inf}$), AUC from time 0 to 24 h ($AUC_{24}$), and maximum plasma concentration ($C_{max}$). For the MAD cohort, the parameters were $AUC_{24}$ and $C_{max}$ on day 1, plus AUC to the end of the dosing period ($AUC_{tau}$) and $C_{max}$ on day 14 or 17.

The statistical linear relationship between the ln-transformed PK parameters and the ln-transformed dose was verified by including the quadratic $(\ln \text{Dose})^2$ and cubic $(\ln \text{Dose})^3$ effects. The statistical linear relationship was established if the quadratic and cubic effects were not statistically significant, using a 5% level of significance, or if the effects were statistically significant, but of such small magnitude that they were not clinically relevant. The dose-proportionality analysis was performed using SAS® PROC MIXED.

If a statistical linear relationship was shown and if the 2-sided 95% CIs around the slope estimate parameters included the value of 1 for dose-dependent parameters, then dose proportionality was established.

*Steady-state analysis*

In the MAD cohort, a steady-state analysis for aficamten was performed on the ln-transformed plasma trough concentration ($C_{trough}$) values using Helmert contrasts. An analysis of variance (ANOVA) model was conducted separately for each dose level; day was included as the fixed effect. Helmert contrasts were developed such that each time-point was compared with the mean of the subsequent time-points. Steady state was established at the time-point where no statistical difference (alpha = 5%, 2-sided) was observed with the subsequent time-points.

**Statistical Analysis of Pharmacodynamic (PD) Parameters**

*Dose–response analysis*

To analyze the impact of drug dose on echocardiographic parameters in the SAD and MAD cohorts, an inferential analysis was conducted on the PD analysis set using linear mixed models for repeated-measures analysis of covariance (ANCOVA). The ANCOVA used baseline value as a covariate, included treatment, time-point, and time-point-by-treatment interaction as fixed effects, and change from baseline as the dependent variable. The unstructured variance-covariance structure was used, and the model accounted for the time-point repeated measures. The ANCOVA analysis was conducted separately for each study part and for each PD parameter. The least-squares means, the difference in least squares means (active minus placebo), and the associated 2-sided 95% CIs were presented for each comparison.

*Concentration bin analysis*

An additional inferential analysis was performed in the SAD and MAD cohorts, to evaluate the relationship between concentrations of aficamten and LVEF for participants in the PK/PD analysis set. A concentration bin ANCOVA was conducted using linear mixed models for repeated-measures analyses with concentration bin group as a fixed effect, baseline PD parameters as a covariate, change from baseline as the dependent variable, and a random intercept to adjust for the repeated measures. The unstructured variance-covariance structure was used. Plasma concentrations of aficamten were paired with coincident PD parameters. The ANCOVA compared the change in PD parameters between each bin versus the pooled placebo group. The least-squares means, the difference in least squares means (bin group minus placebo), and the associated 2-sided 95% CIs were presented for each comparison. The ANCOVA analysis

was conducted separately for each study part. For all time-points at which both PK data and PD measures were available, the time-points were pooled for the analysis. In each part of the study, aficamten concentrations with time-matched PD data were pooled and sorted in increasing order. From least to greatest, the data were then divided into 5 groups of observations ('bins'), each consisting of 20% of the data points. Each bin was treated as a separate group. Concentration bins consisted of a placebo group and 5 bin groups based on the pool of concentrations from all time-points on aficamten treatment.

*Exposure–response trend*

The above analysis was then repeated using concentration as a continuous variable to estimate the exposure–response trend. Both a random intercept effect and a random concentration effect were added to the ANCOVA. The estimate of the concentration slope, with corresponding 2-sided 95% CIs, and the ANCOVA analyses were presented for each study part.

**SUPPLEMENTAL TABLE 1: Echocardiogram-Related AEs of Decreased Ejection Fraction <45%**

| Cohort, Participant | Dose | Grade | Onset* | LVEF Deemed AE[†] | Outcome[‡] |
|---|---|---|---|---|---|
| SAD, 42-year-old white female | 40 mg | 1 (mild) | 1.4 h | 42.2% | Resolved 4.6 h later (6 h after drug dose), with LVEF recorded at 58.4% |
| SAD, 46-year-old white male | 50 mg | 1 (mild) | 1.5 h | 42.1% | Resolved 2.5 h later (4 h after drug dose), with LVEF recorded at 50.2% |
| SAD, 36-year-old white/Hispanic male | 75 mg | 1 (mild) | 1.5 h | 34.6% | Resolved 2.5 h later (4 h after drug dose), with LVEF recorded at 51.9% |

*Time after drug dose.

[†]LVEF values for safety assessments were determined by the study cardiologist.

[‡]In the SAD cohorts, post-dose echocardiograms were performed at 1.5, 4, 6, 24, and 48 h post dose.

AE = adverse event; LVEF = left ventricular ejection fraction; SAD = single-ascending dose.

**SUPPLEMENTAL TABLE 2: Summary of Plasma Aficamten Pharmacokinetics in the CYP2D6 Poor Metabolizer Cohort**

| PK Parameter | CYP2D6 Poor Metabolizers (n = 7) |
|---|---|
| $C_{max}$ (ng/ml) | 57.3 (55.0) |
| $T_{max}$ (h) | 1.0 (0.5, 4.0) |
| $AUC_{24}$ (ng·h/ml) | 495 (19) |
| $AUC_{inf}$ (ng·h/ml) | 2966 (46) |
| $t_{½}$ (h) | 110.2 ± 47.3 |
| CL/F (L/h) | 3.6 ± 1.4 |
| Vz/F (L) | 506.0 ± 84.4 |

Aficamten dosage was 10 mg.

AUC and $C_{max}$ values are presented as geometric mean and geometric CV%, $T_{max}$ values as median (range), and all other parameters as arithmetic mean ± standard deviation.

$AUC_{24}$ = area under the plasma drug concentration–time curve from time 0 to 24 h; $AUC_{inf}$ = AUC from time 0 extrapolated to infinity; CL/F = apparent total body clearance; $C_{max}$ = maximum plasma concentration; CV% = percent coefficient of variation; PK = pharmacokinetic; $t_{½}$ = half-life; $T_{max}$ = time to maximum plasma concentration; Vz/F = apparent volume of distribution.

**SUPPLEMENTAL TABLE 3: Summary of Plasma Aficamten Pharmacokinetics in the Food-Effect Cohort**

| PK Parameter | Fasted (n = 10)* | Fed (n = 12)† |
|---|---|---|
| $C_{max}$ (ng/ml) | 50.6 (47.2) | 65.0 (69.5) |
| $T_{max}$ (h) | 2.3 (0.6, 6.0) | 1.5 (0.5, 6.0) |
| $AUC_{24}$ (ng·h/ml) | 601 (33) | 631 (25) |
| $AUC_{inf}$ (ng·h/ml) | 3674 (24) | 3413 (32) |
| $t_{½}$ (h) | 84.4 ± 16.4 | 78.0 ± 8.1 |
| CL/F (L/h) | 2.8 ± 0.6 | 3.1 ± 0.9 |
| Vz/F (L) | 345.7 ± 133.7 | 339.7 ± 100.4 |

Aficamten dosage was 10 mg in each cohort.

*n = 4 for $AUC_{inf}$, $t_{½}$, CL/F, and Vz/F.

†n = 7 for $AUC_{inf}$, $t_{½}$, CL/F, and Vz/F.

AUC and $C_{max}$ values are presented as geometric mean and geometric CV%, $T_{max}$ as median (range), and all other parameters as arithmetic mean ± standard deviation.

$AUC_{24}$ = area under the plasma drug concentration–time curve from 0 to 24 h; $AUC_{inf}$ = AUC from time 0 extrapolated to infinity; CL/F = apparent total body clearance; $C_{max}$ = maximum plasma concentration; CV% = percent coefficient of variation; $t_{½}$ = half-life; $T_{max}$ = time to maximum plasma concentration; Vz/F = apparent volume of distribution.

## SUPPLEMENTAL TABLE 4: Echocardiographic Parameters

| | Placebo (n = 15) | | | | | | 50 mg (n = 11) | | | | | | |
|---|---|---|---|---|---|---|---|---|---|---|---|---|---|
| | Baseline | | 1.5 h | | CFB | | Baseline | | 1.5 h | | CFB | | p value |
| | Mean | SD | Mean | SD | Mean | SD | Mean | SD | Mean | SD | Mean | SD | CFB v CFB |
| Heart rate (bpm) | 61.6 | 5.6 | 61.4 | 7.6 | -0.2 | 5.6 | 61.7 | 11.5 | 62.3 | 10.6 | 0.5 | 5.8 | NS |
| LVEF (%) | 66.0 | 2.0 | 65.8 | 2.0 | -0.3 | 1.8 | 66.3 | 3.8 | 60.5 | 7.3 | -5.8 | 6.1 | 0.003 |
| LV ESV (mL) | 32.0 | 5.5 | 31.1 | 5.5 | -0.9 | 3.6 | 31.6 | 6.8 | 39.7 | 10.4 | 8.1 | 8.4 | 0.001 |
| LV EDV (mL) | 93.6 | 15.2 | 90.6 | 14.4 | -3.0 | 8.1 | 93.2 | 13.2 | 99.8 | 13.4 | 6.6 | 10.6 | 0.016 |
| LV SV (mL) | 56.7 | 6.3 | 56.1 | 9.7 | -0.6 | 6.8 | 58.9 | 8.0 | 52.6 | 10.5 | -6.2 | 7.6 | NS |
| LV CO (mL) | 3470 | 299 | 3450 | 655 | -21.4 | 538 | 3580 | 597 | 3240 | 681 | -346 | 498 | NS |
| IVCT (msec) | 46.9 | 2.4 | 46.9 | 6.4 | 0.4 | 5.9 | 42.3 | 1.6 | 42.6 | 1.8 | 0.4 | 2.9 | NS |
| IVRT (msec) | 72.1 | 10.6 | 73.9 | 7.0 | 1.9 | 11.3 | 68.7 | 3.0 | 69.3 | 3.3 | 0.5 | 3.8 | NS |
| LV ET (msec) | 332 | 32.2 | 324 | 26.3 | -6.9 | 12.5 | 344 | 35.1 | 328 | 34.1 | 1.9 | 12.8 | NS |
| LAV (mL) | 36.0 | 8.4 | 35.3 | 9.3 | -1.0 | 7.6 | 35.7 | 9.9 | 35.7 | 7.2 | 0.0 | 4.7 | NS |
| E (cm/sec) | 73.6 | 6.7 | 68.2 | 8.2 | -5.3 | 5.7 | 66.7 | 14.7 | 64.0 | 14.7 | -2.7 | 4.5 | NS |
| A (cm/sec) | 49.4 | 11.4 | 47.7 | 10.3 | -1.7 | 5.6 | 52.0 | 12.8 | 54.5 | 17.5 | 2.5 | 6.6 | NS |
| e' lateral (cm/sec) | 12.7 | 2.5 | 12.3 | 2.7 | -0.7 | 1.5 | 11.5 | 3.1 | 10.2 | 2.4 | -1.3 | 1.6 | NS |
| E/A ratio | 1.6 | 0.4 | 1.5 | 0.4 | -0.1 | 0.2 | 1.3 | 0.1 | 1.2 | 0.2 | -0.1 | 0.1 | NS |
| E/e' ratio | 6.0 | 1.2 | 5.8 | 1.3 | -0.1 | 0.7 | 6.3 | 2.8 | 6.8 | 3.3 | 0.5 | 1.5 | NS |

The table compares the placebo data of echocardiographic parameters at baseline and 1.5 h (the time of the echocardiogram closest to peak of plasma concentrations of aficamten) to those at 50 mg of aficamten (the highest well tolerated single dose).

A = peak A wave velocity, bpm = beat per minute, CFB = change from baseline, CO = cardiac output, E = peak E wave velocity, EDV = end-systolic volume, ESV = end-systolic volume, ET = ejection time, e' lateral = tissue doppler velocity of the lateral wall, IVCT = isovolumic contraction time, IVRT = isovolumic relaxation time, LAV = left atrial volume, LVEF = left ventricular ejection fraction, NS = not significant (p > 0.05), SD = Standard deviation, SV = stroke volume

**SUPPLEMENTAL FIGURE 1: Aficamten Plasma Concentration Over Time With Single Doses of Aficamten**

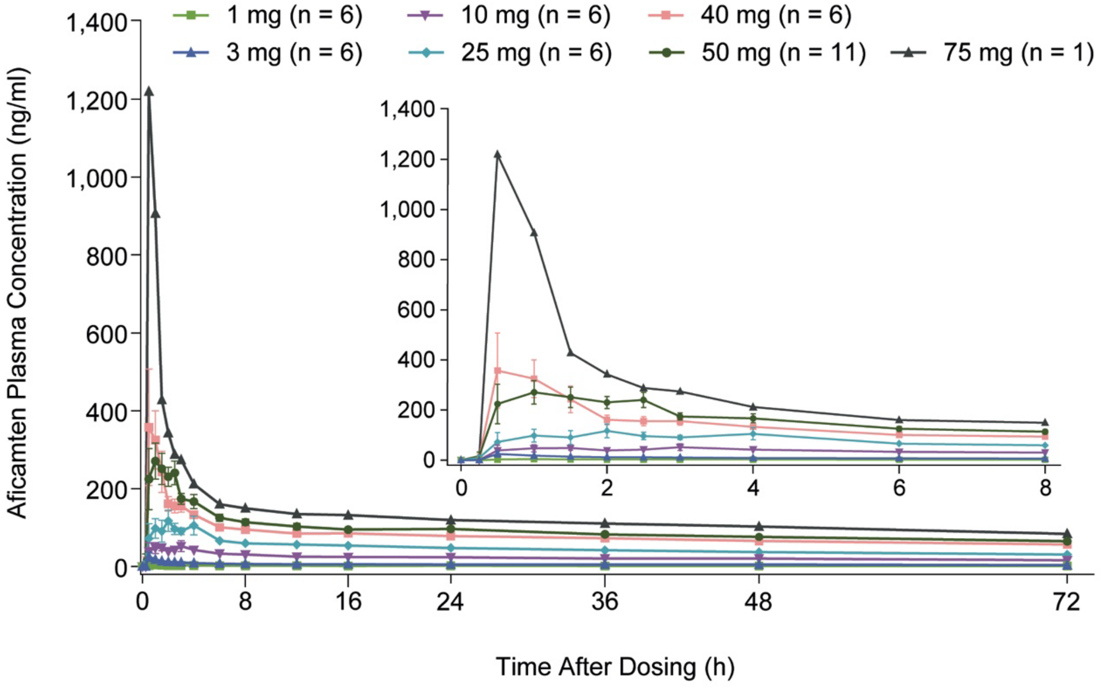


Mean (SE) plasma concentration of aficamten increased in a dose-proportional manner following single oral doses (1, 3, 10, 25, 40, 50, and 75 mg) in healthy participants. Across the doses studied, median time to reach peak concentrations were between 0.50 and 2.76 h post dose. Terminal elimination half-life estimates were consistent across doses.

Inset shows the interval from 0 to 8 h on an extended time scale.

SE = standard error.

**SUPPLEMENTAL FIGURE 2: Plasma Aficamten Concentration Over Time in CYP2D6 Poor Metabolizers**

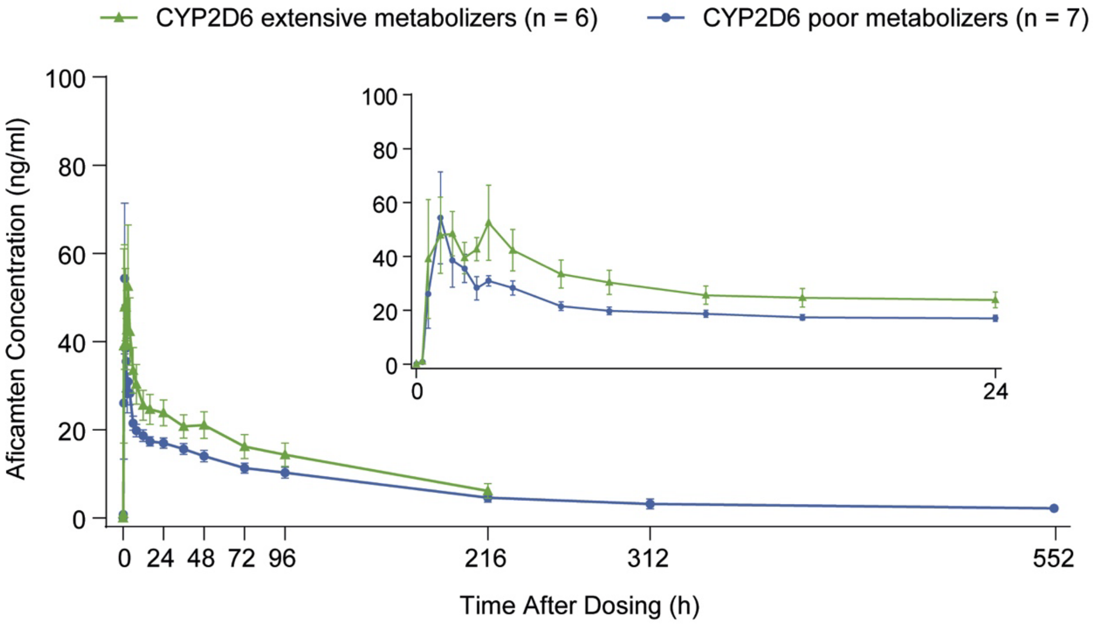


Modestly lower aficamten plasma mean (SE) concentrations were observed in CYP2D6 poor metabolizers versus extensive metabolizers following a single 10-mg dose.

Inset shows the interval from 0 to 24 h on an extended time scale.

SE = standard error.

**SUPPLEMENTAL FIGURE 3: Plasma Aficamten Concentration Over Time Profiles in Fed or Fasted Conditions**

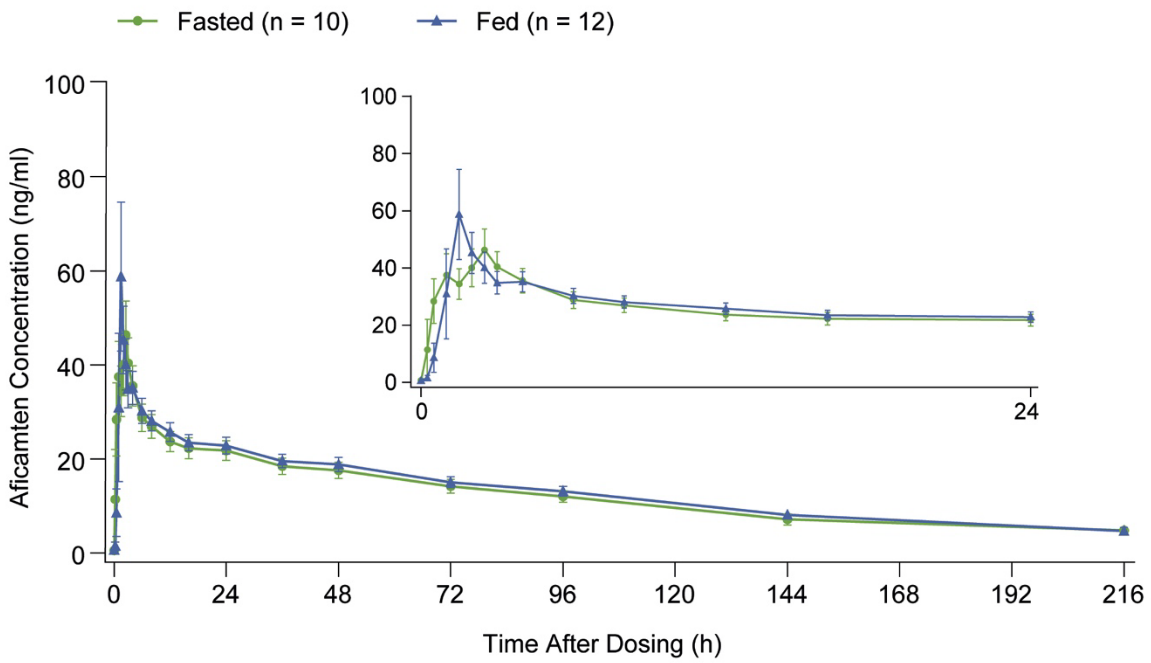


Mean (SE) aficamten plasma concentrations displayed modest overall delay in absorption in fed versus fasted participants following a single 10-mg dose.

Inset shows the interval from 0 to 24 h on an extended time scale.

SE = standard error.
